# Polarimetric imaging of collagen in histopathology specimens: an investigation of congo red and picrosirius red-stained placenta and skin

**DOI:** 10.1038/s41598-026-37711-8

**Published:** 2026-03-06

**Authors:** Georgia Mappa, Pika Miklavc, Michele Cummings, Richard Oliver, Harriet Pyrah, Clare Freer, Huda Alzahrani, Tiehan H. Shen, Nicolas M. Orsi

**Affiliations:** 1 University of Leeds, St. James’s University Teaching Hospital, Leeds, LS9 7TF UK; 2 School of Science, Engineering and Environment, University of Salford, Salford, M5 4WT UK; 3 School of Physics and Astronomy, University of Leeds, Woodhouse Lane, Leeds, LS2 9JT UK; 4 Department of Physics, College of Science, Taif University, P. O. Box 11099, 21944 Taif, Saudi Arabia

**Keywords:** Biological techniques, Medical research, Optics and photonics

## Abstract

Polarisation microscopy is a label-free technique whose tissue architecture profiling through anisotropy remains underinvestigated. In histology, bright-field microscopy and special stains remain the standard for investigating extracellular matrix (ECM) proteins. However, these approaches are qualitative and observer-dependent. By measuring birefringence parameters, quantitative polarisation microscopy (QPM) could offer a more objective alternative. Using dual photoelastic modulators and full Stokes parameter acquisition, QPM was applied to Congo red and picrosirius red-stained sections of human placenta, normal skin, and keloid scars. From the Stokes vectors (*I*, *Q*, *U*, *V*), polarisation azimuth, ellipticity, phase retardation, and depolarisation maps were derived, enabling quantitative characterisation of collagen organisation. QPM revealed distinct anisotropic signatures corresponding to ECM microarchitecture. Picrosirius red-stained sections demonstrated a three/four-fold in phase retardation increase compared to Congo red, confirming its collagen-specific birefringent enhancement. In placenta, spatially heterogeneous birefringence highlighted perivascular collagen arrangements. In skin, stronger phase retardation and depolarisation patterns were observed in dermal collagen bundles, with keloid scars showing markedly increased phase retardation (~ 1.4 radians) and depolarisation (~ 0.96), reflecting altered collagen density and disorganisation. QPM provides objective, spatially-resolved tissue anisotropy metrics, overcoming the qualitative limitations of traditional histological techniques. This approach offers a robust framework for assessing collagen remodelling in various pathological contexts.

**Supplementary Information:**

The online version contains supplementary material available at 10.1038/s41598-026-37711-8.

## Introduction

Polarisation microscopy offers a powerful, label-free modality for investigating tissue architecture by exploiting the anisotropic optical properties of biological molecules. Despite its established use in material sciences and mineralogy, its adoption in clinical histology has been relatively limited. Yet, as an imaging approach sensitive to molecule orientation and organisation in situ, polarisation microscopy is uniquely positioned to reveal subtle structural tissue alterations that may be invisible to, or poorly appreciated by, conventional brightfield microscopy or standard histochemical stains. In histology, bright-field microscopy coupled with special stains (e.g., picrosirius red for collagen) remains the mainstay for accentuating the appearance of extracellular matrix (ECM) proteins. However, these techniques are largely qualitative and observer-dependent, offering limited information on structural alignment or their relative degree of anisotropy. By contrast, polarisation microscopy provides quantifiable data on birefringence, such as phase retardation and depolarisation properties, allowing for an objective assessment of, for example, ECM alterations^1^. Given its potential merit in assessing ECM structural organisation, the integration of polarisation microscopy into tissue assessments could align itself as a tool for histological evaluation and, in the case of pathology, risk stratification and monitoring disease progression^2,3^.

Quantitative polarisation measurements, collectively termed polarimetry, have been well demonstrated across diverse scientific and industrial applications, spanning fields such as astronomy (studying interstellar dust polarisation^4^, remote sensing (vegetation and aerosol characterisation^5^, magneto-optics (Faraday rotation in materials^6^, and ellipsometry (thin-film analysis^7^. Polarimetry is also widely used in industry, particularly for measuring the concentration of optically active compounds (e.g., sugars, pharmaceuticals), which is indispensable in the food, beverage and pharmaceutical sectors^8^. Recently, polarimetric imaging of birefringent crystals in synovial fluid has been reported in a biomedical context using a novel polarised light microscope^9^. Despite these broad applications, systematic use of polarimetric imaging in histology workflows remains limited. Importantly, several advances in quantitative polarisation microscopy have been made in biomedical contexts, particularly with PolScope-based systems. PolScope^10^ employs liquid crystal modulators to extract polarisation information and has been applied to characterise collagen fibre organisation in pancreatic cancer, breast cancer, and other tissues^11–13^. However, while studies have highlighted its ability to discriminate healthy from malignant tissue based on birefringence signatures^14^, its adoption as an investigative histology tool is still emerging^15^.

The present approach differs from PolScope in both design and signal recovery methodology. Whereas PolScope relies on liquid crystal modulation (a ‘direct current’ approach to signal extraction), our dual photoelastic modulator polarimeter employs lock-in detection (an ‘alternating current’ approach), where LED intensity modulation is phase-locked to modulator reference frequencies. We therefore position this work within the context of histology and ECM characterisation, highlighting the complementary potential of advanced Stokes polarimetry to established systems such as PolScope.

A quantitative polarisation microscopy approach could represent a significant advance in this context. Stokes polarimetry measures the complete polarisation state of light, described by the Stokes parameters^16^. These enable quantitative assessment of birefringence, optical phase retardation, and depolarisation. Importantly, this approach can capture depolarisation effects that conventional crossed-polariser systems cannot readily detect. The dual photoelastic modulator-based polarimeter design further enables lock-in signal detection, substantially improving noise rejection and sensitivity, allowing detection of weaker anisotropic signals that may otherwise be missed^17^. This offers several key advantages that could make it valuable for histological investigation. Firstly, it enables an objective and quantitative assessment of molecular anisotropy beyond the inherent subjectivity of qualitative interpretation associated with traditional polarised light microscopy or special stains. Secondly, it enables the direct measurement of depolarisation, a subtle yet significant optical property that reflects microstructural disorder or heterogeneity that is not readily captured by conventional imaging techniques. Finally, the system supports large-area imaging while preserving fine structural resolution, ensuring that detailed information about tissue architecture is retained across extended fields of view - an essential requirement for both research and histology applications.

This study aimed to apply an advanced Stokes polarimetry system to demonstrate its investigative merits, focusing on collagen, an intrinsically anisotropic ECM molecule whose organisation is critical to tissue integrity and function^18,19^. We examined collagen birefringence and depolarisation across (i) placenta, where collagen provides a key structural and functional framework, (ii) normal skin, where collagen fibres are arranged in a different (and thus comparator) histoarchitectural arrangement, and (iii) keloid scars, which are characterised by aberrant collagen deposition (including larger fibre diameter and disrupted architecture)^20^. To further validate our system’s ability to detect and discriminate collagen-specific signals, we compared two commonly used histological stains (Congo red and picrosirius red) since they differ in their molecular interactions with collagen and in the degree of anisotropic enhancement they confer^21–23^. Through these comparative analyses, we aimed to (i) quantify differences in collagen architecture across normal and pathological settings, (ii) illustrate the sensitivity and specificity of our Stokes polarimetry system, and (iii) highlight its potential for studying ECM/collagen remodelling.

## Results

### Polarimetric profiling of placental tissue

**Fig. 1:**
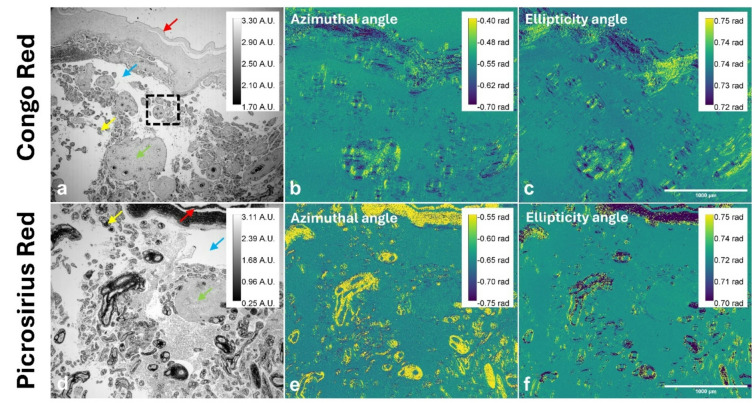
Representative polarimetric imaging of a Congo red-stained placenta section under nearly circularly polarised light illumination using a 4x objective lens. **a** Stokes parameter *I*: total intensity (square inlay indicates area selected for higher resolution imaging shown in Figs. 3 and 4; arrows represent structures of interest: foetal membranes (red), intervillous space (blue), stem villus (green) and terminal villus (yellow)), **b** polarisation azimuthal angle, indicating the orientation of major axis of the polarisation state, and **c** ellipticity angle, representing the degree of circular polarisation at the sample. For comparison, corresponding polarimetric images of a sequential placental section stained with picrosirius red and imaged under identical conditions are shown in (**d**)–(**f**): **d** Stokes parameter *I*, **e** polarisation azimuthal angle and **f** ellipticity angle. Scale bars: 1000 μm. (*A.U.* Arbitrary Units, *rad* radians).

Polarimetric imaging provided quantitative insights into the structural organisation of collagenous components within placental tissue. Figure 1(a) presents the spatial distribution of the Stokes parameter *I*, (*Q*, *U* and *V* provided in Figure S3), for a Congo red-stained placental section under nearly circularly polarised illumination. Figure 1(b) illustrates the variation in polarisation azimuthal angles across the sample, while Fig. 1(c) shows corresponding changes in ellipticity. For comparison, images from a sequential placental section stained with picrosirius red are shown in Fig. 1(d)-(f), with Fig. 1(d) showing the spatial distribution of the Stokes parameter *I*. The polarisation azimuthal angle (Fig. 1(e)) and ellipticity map (Fig. 1(f)) reveal distinct alignment patterns in anisotropic molecular structures indicative of collagen organisation.

**Fig. 2:**
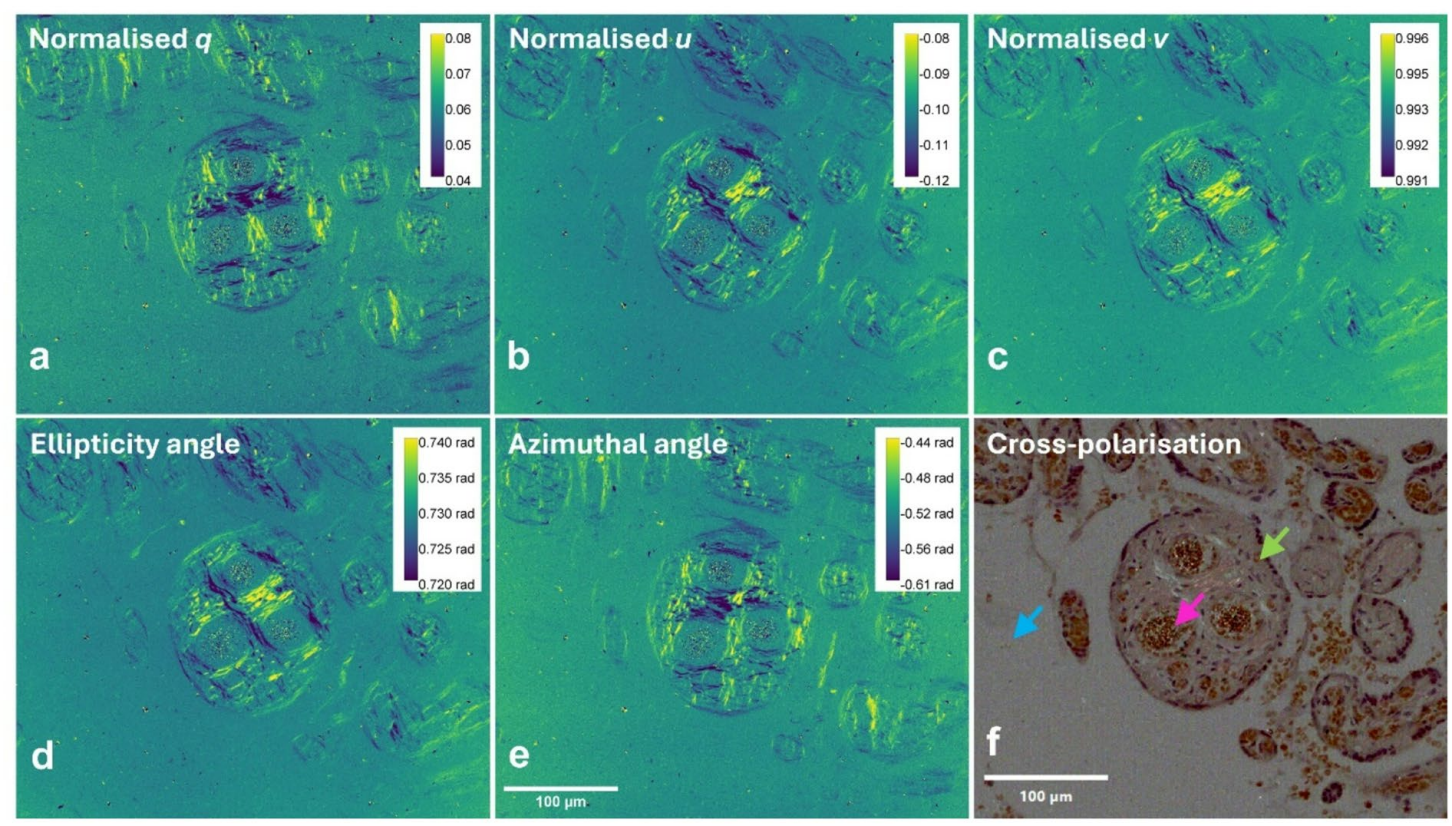
High-magnification polarimetric imaging of a Congo red-stained placenta section corresponding to the square inlay indicated in Fig. 1(a), using a 40x objective lens. **a** Normalised Stokes parameter *q*, **b** normalised Stokes parameter *u*, and **c** normalised Stokes parameter *v*, representing the linear and circular polarisation components after normalisation, **d** ellipticity angle, indicating the degree of circular polarisation, and **e** polarization azimuthal angle, denoting the major axis of the polarisation, **f** cross-polarised image, demonstrating a weak birefringent signature. Arrows represent structures of interest: intervillous space (blue), villus (green) and intravillous vessel (purple). Scale bars: 100 μm. (*rad* radians).

At higher magnification, as shown in Fig. 2(a)-(e), the spatial heterogeneity of polarisation properties became more apparent. Specifically, the normalised Stokes parameter images *q*, *u* and *v* (Fig. 2(a)-(c)) acquired using a 40x objective lens highlight differential polarisation retention across tissue regions, reflecting variations in local light scattering. The corresponding polarisation azimuthal angle and ellipticity angle maps (Fig. 2(d)-(e)) further confirmed this spatial heterogeneity. Additionally, the cross-polariser image (Fig. 2(f)) revealed a weak birefringent signal surrounding the blood vessels, consistent with the features identified in the polarimetric images. Corresponding higher magnification images of picrosirius red stained sample are shown in the Supplementary Materials (Figure S4(d)-(f)).

**Fig. 3:**
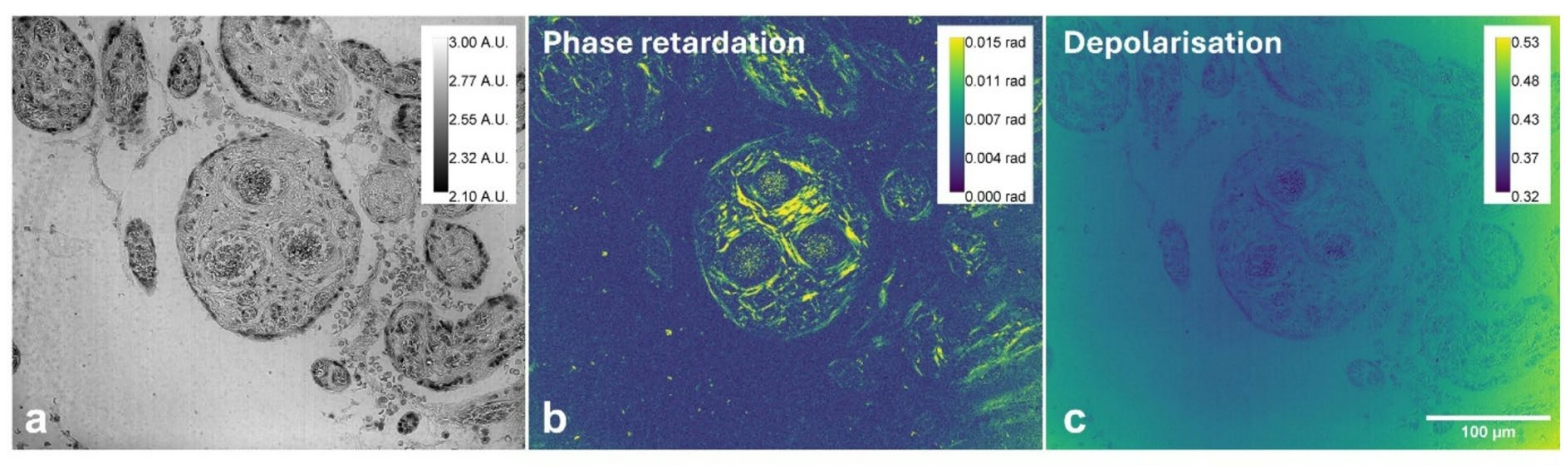
Phase retardation and depolarisation images derived from the initial polarimetric images (I, Q, U, V) acquired under nearly circularly polarised illumination using a 40x objective lens. **a** Intensity image (Stokes parameter *I*), showing the tissue structure of the Congo red-stained placenta section, **b** phase retardation (in radians), and **c** depolarisation image. Scale bar: 100 μm. (*A.U.* Arbitrary Units, *rad* radians).

To further quantify ECM alterations, birefringent phase retardation and depolarisation characteristics were assessed. Figure 3(a) is the brightfield image represented by the intensity (Stokes parameter *I*) showing the overall tissue morphology visible under standard optical microscopy. Figure 3(b) presents a mapping of the phase retardation based on the model described (Eq. (14)). The baseline background of the phase retardation image was 0.11 radians. After background correction, localised regions exhibited negative phase retardation values (relative to the baseline), indicating that the optical axis in these regions was rotated by 90° with respect to the x-axis of the polarimeter coordinates. Figure 3(c) shows the corresponding depolarisation image ($\:{D}_{p}$).

The observed non-uniform phase retardation distribution suggests localised disruptions in collagen crosslinking. The strongest phase retardation, observed around a cluster of three blood vessels, reached approximately 0.04 radians, and was statistically significant in both Congo and picrosirius red when compared to areas with no tissue (*p* < 0.001: Figure S7.1 B).The background depolarisation values ranged between 0.40 and 0.48, where the tissue structure exhibited lower depolarisation (typical value of 0.37 than that of the substrate). Although the substrate appeared to produce marginally higher depolarisation than the tissue, the difference was statistically significant for picrosirius red (*p* < 0.001: Figure S7.1 A). No significant differences in depolarisation or phase retardation were found when comparing Congo and picrosirius red, either in substrate or tissue.

### Comparative polarimetric assessment of normal skin

**Fig. 4:**
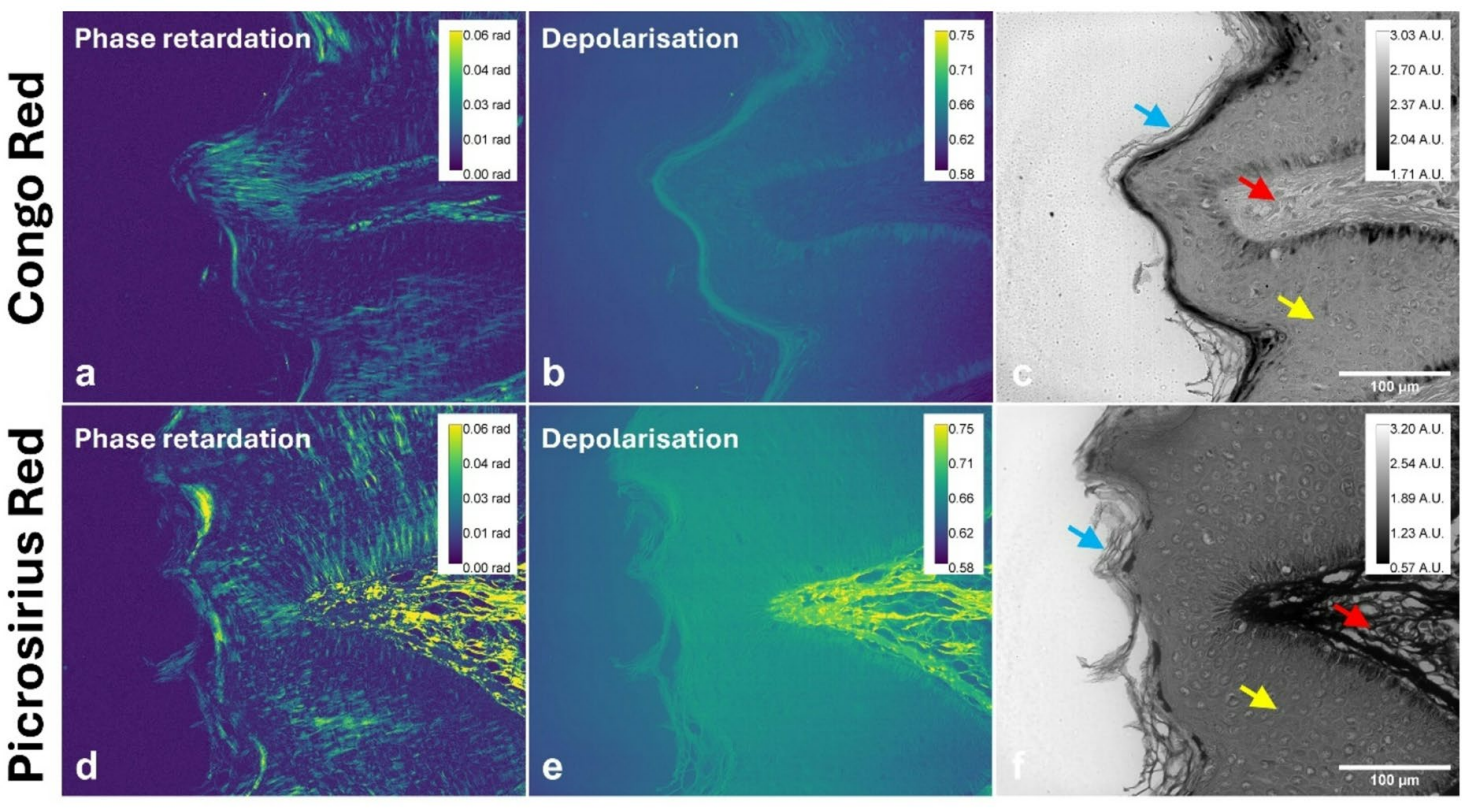
Polarimetric analysis of a Congo red-stained skin section (**a**–**c**) and a sequential one stained with picrosirius red (**d**–**f**): **a** phase retardation within the sample; **b** depolarisation mapping, illustrating polarisation loss; **c** the brightfield image (intensity, Stokes parameter *I*). Polarimetric analysis of a picrosirius red sequential section: **d** the phase retardation image; **e** the depolarisation image and **f** the brightfield image. Arrows indicate tissue structures of interest: surface keratin (blue), epidermis (yellow) and papillary dermis (red). Scale bars: 100 μm. (*A.U.* Arbitrary Units, *rad* radians).

To validate the sensitivity of the polarimetric approach in detecting collagen distribution, placental samples were compared with normal skin, where fibre arrangement was anticipated to follow a non-circular distribution. Figure 4 presents the polarimetric analysis of a Congo red-stained skin sample and a picrosirius red stained sequential section of the same specimen. Figure 4(a) is the phase retardation, Fig. 4(b) the depolarisation image and Fig. 4(c) the brightfield image (intensity, Stokes parameter *I*) for the Congo red stained sample. Figure 4(d)- (f) are the images of the phase retardation, the depolarisation and the brightfield, respectively, for the picrosirius red stained sample.

For Congo red, a significant increase for both depolarisation (*p* = 0.005) and phase retardation (*p* < 0.001) was observed between background (no tissue) and the epidermis (Figure S7.2 A and B). The phase retardation (*p* < 0.001) statistically increased when comparing the papillary dermis to background. The papillary dermis was statistically decreased compared to epidermis for depolarisation (*p* = 0.002). Similarly for picrosirius red a significant increase in both depolarisation and phase retardation between the background and epidermis, as well as between background and the papillary dermis (*p* < 0.001) was observed. A significant increase was also observed between epidermis and papillary dermis for both depolarisation and phase retardation (*p* < 0.001). Of note, the papillary dermis between Congo red and picrosirius red showed a significant increase in depolarisation (*p* = 0.019) and phase retardation (*p* = 0.003). The brightfield images (Figs. 4(c) and (f)) provide morphological context, enabling visual correlation between structural features and polarimetric measurements.

### Quantitative polarimetric comparison of keloid vs. normal dermis

**Fig. 5:**
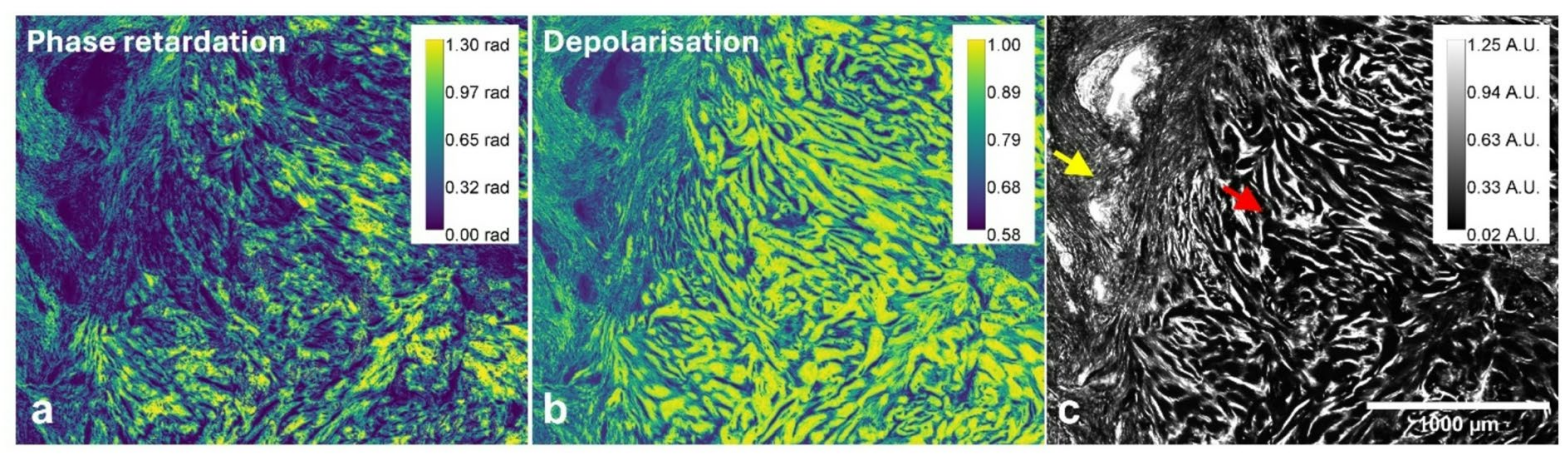
Polarimetric analysis of a picrosirius red-stained skin sample comparing normal and keloid regions. **a** Phase retardation image, illustrating the strength of the birefringence across both normal and keloid regions. **b** depolarisation image, highlighting the differences between normal and keloid regions, **c** the brightfield image (Stokes parameter *I*) for contextual alignment. Arrows indicate tissue structures of interest: keloid (red) and surrounding reticular dermis (yellow). Scale bar: 1000 μm. (*A.U.* Arbitrary Units, *rad* radians).

Figure 5 provides a direct comparison between the keloid region and surrounding normal reticular dermis. The phase retardation map (Fig. 5(a)) and depolarisation map (Fig. 5(b)), clearly delineate the increased collagen density and altered fibrillar organisation in keloid tissue. In the normal region (see arrows in Fig. 5(c)), the phase retardation values were at most 1.00 rad, whilst in the keloid region the phase retardation was approximately 1.40 radians. The average depolarisation was statistically increased (*p* = 0.002) in the keloid region when compared to the normal region (Figure S7.3 A and B). The maximum depolarisation in the keloid region was about 0.96, indicating extensive depolarisation in the lesion. The pronounced birefringence and strong depolarisation in the keloid region relative to surrounding reticular dermis validates the efficacy of this imaging approach in highlighting aberrant ECM remodelling.

## Discussion

This study demonstrates the value of quantitative polarisation microscopy (QPM) as a label-free and quantitative tool for investigating ECM architecture in histological specimens. By measuring the full Stokes parameters, phase retardation, and depolarisation, our approach extends beyond the qualitative capabilities of conventional polarised light microscopy and special stains, offering an objective and spatially resolved characterisation of anisotropic molecular structures. The Stokes parameters (*I*, *Q*, *U*, *V*) capture the raw polarisation state at each pixel, whereas normalised parameters (*i*, *q*, *u*, *v*) isolate polarisation effects by removing intensity variation. Parameters *q* and *u* represent linear polarisation, and *v* represents circular polarisation. Thus, a non-zero *v* from initially linearly polarised light signals sample birefringence. From these, ellipticity and azimuthal angles can be derived, reflecting both the input light and sample-induced polarisation changes. Phase retardation instead measures birefringence directly, highlighting structural anisotropy relevant to histology. Using a birefringence model with nominally circularly polarised input (ellipticity ≈ 41°), we obtained robust results. QPM therefore offers a more objective assessment of anisotropy than traditional cross-polariser microscopy. As such, unlike conventional crossed-polarisation systems which can only visualise aligned fibres, QPM enables direct measurement of depolarisation—a subtle but important property reflecting microstructural disorder^2,15^. The use of dual photoelastic modulators and lock-in detection improves signal-to-noise ratio, allowing the detection of weaker anisotropic signals that might be missed by traditional methods^17^. Importantly, the system provides quantitative, observer-independent data that could support automated analysis and threshold-based classification in histology/pathology.

The tissues examined were selected for their expected differences in collagen fibre arrangement and size: circular and perivascular distribution in placental villi^24^, more layered and mesh-like bundles in dermis^18,19^, and aberrantly thickened, haphazard in keloid scars^25^. The quantitative polarisation data were broadly in line with these expectations. In placenta, low but spatially heterogeneous birefringence and depolarisation were observed corresponding to stromal collagen around villi. In particular, collagen fibres were seen to surround villous vessels in a concentric arrangement. By contrast, in skin reticular dermis, phase retardation and depolarisation were much more pronounced and highlighted dermal collagen bundles. While the observation of higher retardation values likely indicates thicker collagen fibres or bundles, the strong depolarisation also suggests that the collagen bundles may contain micron-scale substructures or clusters which cause depolarisation scattering. Moreover, the structure of superficial skin contained two different anisotropic signatures which corresponded to epidermal surface keratin and papillary dermal collagen, respectively, in line with previously published observations^26^. However, while the phase retardation and depolarisation mapping captured the keratinised stratum corneum’s contribution, the dominant birefringence arose from deeper collagen-rich dermis given its higher density and thicker fibre bundles^18^. Keloid regions showed even more markedly increased birefringence and depolarisation, reflecting increased collagen fibre size, density and disorganisation^27^.

Comparing Congo red and picrosirius red staining, samples stained with the latter were seen to give rise to a phase retardation signal 3 or 4 times stronger than that of Congo red stained samples, indicating the markedly different degrees of anisotropic enhancement associated with each stain. This also aligns with picrosirius red’s known ability to enhance collagen birefringence due to its linear alignment along collagen fibrils and strong dichroic properties^21^. Congo red, while also birefringent, is not as specific for collagen and can bind to other molecules (e.g., amyloid and a wider array of ECM proteins), resulting in lower enhancement^22^. This justifies its histological use for the clinical detection of amyloid deposits^23^ rather than collagen.

Beyond collagen, QPM could still be applied to address histopathological questions. This methodology could be of value in detecting amyloid deposits^28^ or in assessing the dermal collagen architectural disruption which occurs in early cutaneous squamous cell carcinoma^29^. In the latter case, for example, one of the challenges faced by histopathologists includes the identification of areas of microinvasion in squamous cell carcinoma in situ. In this setting, quantitative assessment of disruptions in dermal collagen may aid the detection of such foci. Other potential applications of quantitative metrics may also help characterise ECM changes in inherited connective tissue disorders such as those occurring in Ehlers–Danlos syndromes^30^ or in photoaged skin (solar elastosis)^31^. These applications would benefit from the technology’s large-area imaging capability while retaining fine spatial resolution.

This study has a number of limitations. Firstly, changes in polarisation state may originate from multiple anisotropic molecules (e.g., collagen, elastin, keratin and amyloid), which may swamp another’s signal based on prevalence/amount, thereby complicating target-specific interpretation of different anisotropic molecules. A multimodal microscopy approach may mitigate this limitation. The polarimetric imaging methodology employed in this study is compatible with other optical microscopy methods. In principle, the polarimetric imaging functionality may be integrated into a microscope of different modality. For example, a combination of polarimetric imaging and fluorescence microscopy would allow pixel-matched images of phase retardation and fluorescence mapping to be collated, identifying areas of structural anisotropy and their underlying molecular origin. Secondly, the sensitivity of birefringence to fibre thickness and alignment means that absolute values may vary with the orientation of sectioned tissue. Thirdly, although the aim of this study was to demonstrate the merit of QPM in this setting, true high-throughput application would require automation of acquisition and analysis, and robust correction for background depolarisation. In this respect, an ideal QPM system would include multi-wavelength capability, higher spatial resolution, and real-time imaging to better differentiate overlapping anisotropic components^32^. Fourthly, the platform’s current image acquisition time is circa 60 s, a parameter principally limited by the camera frame rate. However, this could potentially be reduced to achieve video frame rate with real-time image processing by combining a high-speed camera with a high-performance computer with dedicated graphics card and extensive memory. Finally, variations in sample processing, fixation and staining may impact reproducibility such that large-scale validation would be needed to establish reliable diagnostic thresholds.

In terms of future directions, the quantitative aspects of QPM offer great promise in establishing reproducible thresholds for birefringence and depolarisation that could support diagnostic algorithms. In this regard, integration with machine learning may allow automated detection of microarchitectural changes invisible to human observers^33^. In particular, expanding the remit covered by studies using PolScope in the disruption of collagen fibre organisation in malignancy is a logical next step^11–13^. Furthermore, combined multimodal imaging (e.g., QPM with second harmonic generation microscopy or optical coherence tomography) could further enhance structural insight^34^. Finally, our preliminary data indicate that unstained tissue section collagen signals are detectable; further investigations will focus on this aspect in terms of developing a truly ‘label-free’ application.

In summary, QPM combines visual and quantitative polarisation information, offering detailed characterisation of ECM histoarchitecture across both normal and pathological tissues. This approach may complement existing histological tools, enabling objective analysis and potentially supporting risk stratification or disease monitoring.

## Methods

### Sample preparation and histological staining

This work was conducted under ethical approvals 18/WA/0222 (Wales Research Ethics Committee 5) and 18/LO/0067 (London-Riverside Research Ethics Committee). All experiments were performed in accordance with relevant guidelines and regulations. Informed consent was obtained as appropriate. Human placenta, normal skin and keloid were formalin-fixed and paraffin-embedded (FFPE) specimens from the histopathology archive at St James’s University Hospital, Leeds, surplus to diagnostic requirements. These were chosen due to their anticipated difference in distribution and size of collagen fibres: circular in placental villi, more mesh-like in dermis and larger and more chaotically arranged in keloids. Keloids are a dysregulated scar response, in which increased fibroblast proliferation and decreased apoptosis lead to increased collagen deposition resulting from dysfunctional cytokine (e.g., transforming growth factor (TGF)-β and platelet derived growth factor (PDGF)) production^27^.

Five micron serial sections from each sample were cut using a Leica RM2235 microtome. Superfrost-plus slides (Solmedia, London, UK) were used to mount sections. Slides were incubated at 37 °C for a minimum of 12 h before further processing. For histological staining, sections were deparaffinised and rehydrated through a series of xylene and graded alcohol washes and subsequently stained in Congo red (CR) or picrosirius red (PSR), two special stains commonly used in histology to accentuate collagen or increase birefringence (e.g., for detecting amyloid deposits).

Briefly, slides were placed in CR solution (0.5% Congo red (SLS, UK) in 50% alcohol) for 30 min and rinsed with water before being differentiated in alkaline alcohol (1% sodium hydroxide (Sigma-Aldrich, UK) in 50% alcohol). Sections were rinsed again with water for 1 min before counterstaining with haematoxylin (Sigma-Aldrich) for 30 s. Sections were finally rinsed with water before being dehydrated and mounted using dibutyl phthalate xylene (DPX (Sigma-Aldrich)) with 24 × 40 mm cover slips (Solmedia). For PSR, sections were counterstained with Weigert’s haematoxylin (Atom Scientific, UK) for 8 min. Slides were washed for 10 min in running water and stained in PSR (0.1% Series red F3BA in saturated aqueous solution of picric acid (BDH Chemicals, UK) for 1 h. Sections were finally washed in two changes of acidified water (0.5% acetic acid (Sigma-Aldrich) in H_2_O), dehydrated in 100% ethanol, cleared in xylene (Fisher Scientific, UK) and mounted, as previously described. Orthogonal validation of collagen identity was confirmed using immunohistochemistry (see Figure S6, provided in Section S.6 of the Supplementary Materials).

### Imaging methodology and calibration

Stokes parameters are denoted as *I*, *Q*, *U*, *V*, where *I* is intensity, *Q* the difference in intensity of the linear polarised components along two orthogonal directions, *U* difference in intensity of the linear polarised components along two orthogonal directions which is rotated by 45° with respect to that used for *Q*, and *V* is the difference in intensity of the left and right hand circular polarised components.

**Fig. 6:**
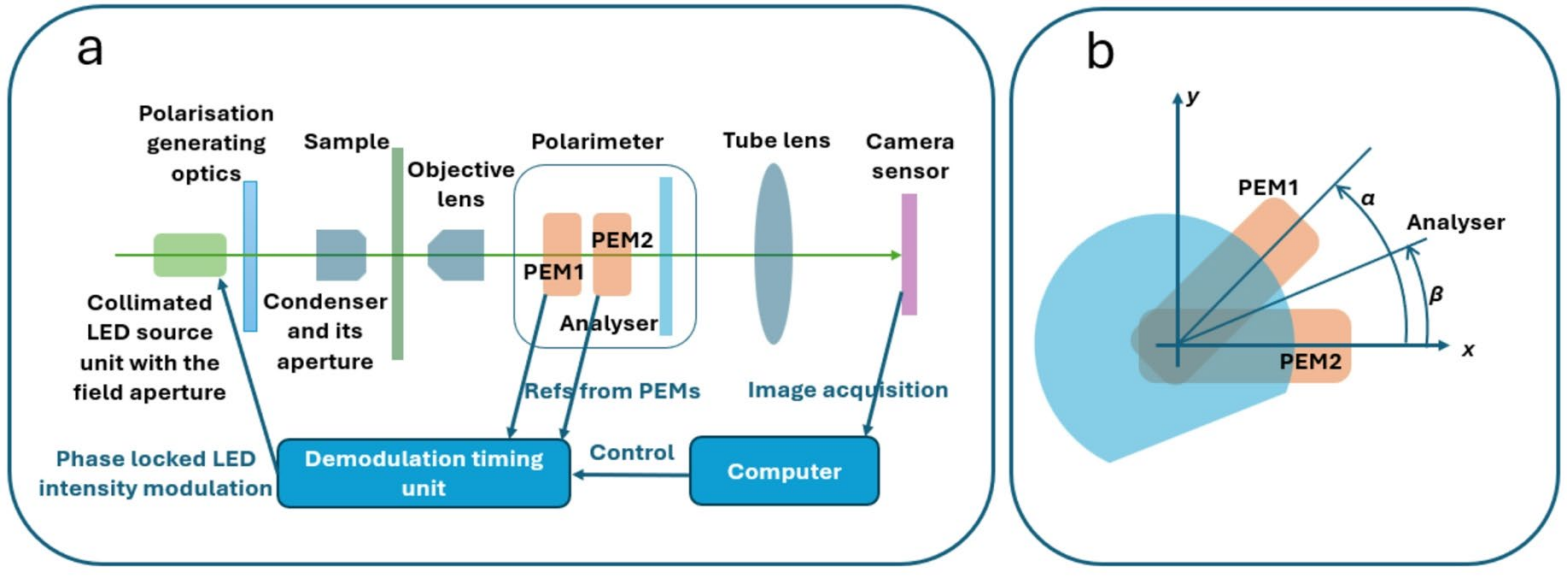
**a** Components of the dual-PEM based Stokes polarimetric microscope. **b** When viewed along the direction of light propagation, the polarimeter configuration establishes a coordinate system in which the optical axis of PEM2 defines the x-axis. The angle between the optical axis of PEM1 and the x-axis is denoted by *α*, while the angle between the transmission axis of the analyser and the x-axis is denoted by *β*. In the present setup, *α* is approximately 45º and *β* is approximately 23º. For the coordinates indicated in (b), the light is propagating along minus *z* direction (i.e., into the page).

Polarimetric imaging was performed using a polarimetric microscope^35^, designed to measure Stokes parameters, thereby the full state of polarisation at a pixel level. The prototype microscope (Fig. 6) employed light emitting diode (LED; M530L4-C1, Thorlabs) illumination for its cost effectiveness and versatility compared to the earlier implementation which utilised a pulsed solid state laser^35^. The polarimetric module, integrated into the infinity space of the infinity-corrected optical path of an Olympus IX-71 inverted microscope (positioned between the objective and tube lenses), comprised two photoelastic modulators (PEMs; I/FS50-ARC-2 and II/FS42-ARC-2 optical heads with PEM-200 controllers, Hinds Instruments) and a linear polarisation analyser (high performance glass linear polariser, Edmund Optics). The PEMs modulate the phase of the incident light, shifting polarisation-dependent signals to harmonics of their resonant frequencies, which enables the separation of the original Stokes components through lock-in detection. A custom demodulation timing unit (DTU) (developed in-house) synchronised the LED intensity modulation with that of the PEMs, allowing phase-sensitive signal recovery at each pixel using a commercial digital camera (Zyla 5.5, Andor) with a maximum sampling rate of 100 Hz. This approach facilitated quantitative polarisation imaging by isolating direct current (DC) and harmonic components which are associated with the Stokes parameters via a 4$\:\times\:$4 transformation matrix *K*^36^. The methodology assumes that the detection system responds linearly to the input light intensity. This assumption has been verified, as demonstrated in the Supplementary Materials (Figure S1), which shows that the camera exhibits a linear response under the microscope setup.

The polarimeter was calibrated in situ using collimated LED sources with a polarisation state generator and the objective lens removed. For this study, the signals included the DC signal ($\:{S}_{DC}$), the second harmonic signals corresponding to the first and second PEM signals (PEM ($\:{S}_{\mathrm{Q}\mathrm{U}1}$) and PEM ($\:{S}_{\mathrm{Q}\mathrm{U}2}$), respectively), and the first harmonic signal of the first PEM ($\:{S}_{\mathrm{v}})$. These signals are mathematically linked to the Stokes parameters (*I*, *Q*, *U*, *V*) through the transformation matrix *K*:

1 $$\:\left(\begin{array}{c}I\\\:Q\\\:U\\\:V\end{array}\right)\begin{array}{cc}=K\left(\begin{array}{c}{S}_{DC}\\\:{S}_{\mathrm{Q}\mathrm{U}1}\\\:{S}_{\mathrm{Q}\mathrm{U}2}\\\:{S}_{\mathrm{v}}\end{array}\right)\end{array}$$

where *K* is defined as:

2 $$\:K=\begin{array}{cc}\left(\begin{array}{cccc}{k}_{1}&\:{k}_{2}&\:{k}_{3}&\:0\\\:0&\:{k}_{4}&\:{k}_{5}&\:0\\\:0&\:{k}_{6}&\:{k}_{7}&\:0\\\:0&\:0&\:0&\:{k}_{8}\end{array}\right)\end{array}$$

and *k*_*i*_ (*i* = 1,…8) are the nonzero elements of *K*. The form of *K* in Eq. (2) is a necessary condition for *K* to exist. In a calibration process, different polarisation states were applied to determine *k*_*i*_ experimentally using a non-linear least square algorithm^35^. The angular configuration of the PEMs and the linear polarisation analyser was chosen to ensure that the determined elements in *K* were experimentally robust. An example of the calibration results is shown in the Supplementary Materials (Figure S2). Once the Stokes parameters are determined, other polarisation characteristics, such as the ellipticity angle *ε*, polarisation azimuthal angle *θ* and the degree of polarisation *P* can be derived using the following equations:

3 $$\:\epsilon\:=\frac{1}{2}\mathrm{arcsin}\left(\frac{V}{\sqrt{{Q}^{2}+{U}^{2}+{V}^{2}}}\right)$$

4 $$\:\theta\:=\frac{1}{2}\mathrm{arctan}\left(\frac{U}{Q}\right)$$

For 100% polarised light, the Stokes parameters satisfy $\:{I}^{2}={Q}^{2}+{U}^{2}+{V}^{2}$. For partially polarised light, $\:{I}^{2}>{Q}^{2}+{U}^{2}+{V}^{2}$. The depolarisation, $\:{D}_{p}$, is therefore defined as:

5 $$\:{D}_{p}=\sqrt{1-{P}^{2}},$$

where

6 $$\:P=\frac{\sqrt{{Q}^{2}+{U}^{2}+{V}^{2}}}{I}\:,$$

is the degree of polarisation of light.

For high-magnification polarimetric imaging in this study, an Olympus 40x polarisation-preserving objective lens (numerical aperture 0.75) was employed. At lower magnification, a standard 4x objective lens (numerical aperture 0.10) was used. The pixel size in the camera was 6.5 μm, and the equivalent sizes for the 40X and 4X objective lenses were 6.17 pixel/µm and 0.617 pixel/µm, respectively. Accordingly, the dimensions of the fields of view (FOV) used for quantitative analysis were 415 × 350µm^2^ and 4.15 × 3.50mm^2^, respectively. The microscope resolution was diffraction limited to approximately 0.3 μm. A 530 nm LED was used as the illumination source. A combination of a polariser (high performance glass linear polariser, Edmund Optics) and a quarter waveplate (WPQ20ME-514, Thorlabs) created a nearly circularly polarised light with an ellipticity angle of about 41° at 530 nm.

### Polarimetric imaging protocol for histopathological applications

The incident light has a polarisation state represented by *i*, *q*, *u*, *v*, while the light exiting the sample has a polarisation state given by $\:{i}^{{\prime\:}}$, $\:{q}^{{\prime\:}}$, $\:{u}^{{\prime\:}}$, $\:{v}^{{\prime\:}}$, where the normalised Stokes parameters are defined as *i = I/I*_*p*_, *q* = *Q*/*I*_*p*_, *u* = *U*/*I*_*p*_ and *v* = *V*/*I*_*p*_, where $\:{I}_{p}=\sqrt{{Q}^{2}+{U}^{2}+{V}^{2}}$, applicable to any none zero polarisation states. Similarly, the corresponding parameters for the transmitted light are $\:{i}^{{\prime\:}}={I}^{{\prime\:}}/{I}_{p}^{{\prime\:}},\:q{\prime\:}=Q{\prime\:}/{I}_{p}^{{\prime\:}}\:$, $\:u{\prime\:}=U{\prime\:}/{I}_{p}^{{\prime\:}}\:\:\:$ and $\:v{\prime\:}=V{\prime\:}/{I}_{p}^{{\prime\:}}.\:$The Stokes parameters *I*, *Q*, *U*, *V* and $\:{I}^{{\prime\:}}$, $\:{Q}^{{\prime\:}}$, $\:{U}^{{\prime\:}}$, $\:{V}^{{\prime\:}}$ for the incident and transmitted light, respectively, were directly measured using the polarimetric microscope with and without the sample.

Consider a birefringent sample of uniaxial anisotropy with phase retardation$\:\:{\delta\:}_{s}$ and its optical axis in the plane of the sample, its associated Mueller matrix $\:{M}_{s}$ can be written as:

7 $$\:{M}_{s}=\begin{array}{cc}\left(\begin{array}{cccc}\sigma\:&\:0&\:0&\:0\\\:0&\:\mathrm{cos}\left(4\gamma\:\right){\mathrm{sin}}^{2}\left(\frac{{\delta\:}_{s}}{2}\right)+{\mathrm{c}\mathrm{o}\mathrm{s}}^{2}\left(\frac{{\delta\:}_{s}}{2}\right)&\:\mathrm{sin}\left(4\gamma\:\right){\mathrm{sin}}^{2}\left(\frac{{\delta\:}_{s}}{2}\right)&\:-\mathrm{sin}\left(2\gamma\:\right)\mathrm{s}\mathrm{i}\mathrm{n}\left({\delta\:}_{s}\right)\\\:0&\:\mathrm{sin}\left(4\gamma\:\right){\mathrm{sin}}^{2}\left(\frac{{\delta\:}_{s}}{2}\right)&\:-\mathrm{cos}\left(4\gamma\:\right){\mathrm{sin}}^{2}\left(\frac{{\delta\:}_{s}}{2}\right)+{\mathrm{c}\mathrm{o}\mathrm{s}}^{2}\left(\frac{{\delta\:}_{s}}{2}\right)&\:\mathrm{cos}\left(2\gamma\:\right)\mathrm{s}\mathrm{i}\mathrm{n}\left({\delta\:}_{s}\right)\\\:0&\:\mathrm{sin}\left(2\gamma\:\right)\mathrm{s}\mathrm{i}\mathrm{n}\left({\delta\:}_{s}\right)&\:-\mathrm{cos}\left(2\gamma\:\right)\mathrm{s}\mathrm{i}\mathrm{n}\left({\delta\:}_{s}\right)&\:\mathrm{c}\mathrm{o}\mathrm{s}\left({\delta\:}_{s}\right)\end{array}\right)\end{array}$$

where $\:\gamma\:$ is the angle between the optical axis of the sample and the *x*-axis of the polarimeter, and $\:{\delta\:}_{s}$ is the phase retardation of the sample^37^. The parameter *σ* is introduced to describe any depolarisation effect of the sample, where $\:\sigma\:={P}^{-1}$.

The relationship between the Stokes parameters is expressed as:

8 $$\:\left(\begin{array}{c}{i}^{{\prime\:}}\\\:{q}^{{\prime\:}}\\\:{u}^{{\prime\:}}\\\:{v}^{{\prime\:}}\end{array}\right)={M}_{s}\left(\begin{array}{c}i\\\:q\\\:u\\\:v\end{array}\right)$$

This leads to the following equations:

9 $$\:{q}^{{\prime\:}}=\left(\left(\mathrm{c}\mathrm{o}\mathrm{s}(4\gamma\:\right){\mathrm{sin}}^{2}\left(\frac{{\delta\:}_{s}}{2}\right)+{\mathrm{c}\mathrm{o}\mathrm{s}}^{2}\left(\frac{{\delta\:}_{s}}{2}\right)\right)q+\left(\mathrm{sin}\left(4\gamma\:\right){\mathrm{s}\mathrm{i}\mathrm{n}}^{2}\left(\frac{{\delta\:}_{s}}{2}\right)\right)u+\left(-\mathrm{s}\mathrm{i}\mathrm{n}\left(2\gamma\:\right)\mathrm{s}\mathrm{i}\mathrm{n}\left({\delta\:}_{s}\right)\right)v\:$$

10 $$\:{u}^{{\prime\:}}=\left(\mathrm{sin}\left(4\gamma\:\right){\mathrm{s}\mathrm{i}\mathrm{n}}^{2}\left(\frac{{\delta\:}_{s}}{2}\right)\right)q+\left(-\mathrm{cos}\left(4\gamma\:\right){\mathrm{sin}}^{2}\left(\frac{{\delta\:}_{s}}{2}\right)+{\mathrm{c}\mathrm{o}\mathrm{s}}^{2}\left(\frac{{\delta\:}_{s}}{2}\right)\right)u+\left(\mathrm{cos}\left(2\gamma\:\right)\mathrm{s}\mathrm{i}\mathrm{n}\left({\delta\:}_{s}\right)\right)v$$

11 $$\:{v}^{{\prime\:}}=\left(\mathrm{sin}\left(2\gamma\:\right)\mathrm{sin}\left({\delta\:}_{s}\right)\right)q+\left(-\mathrm{cos}\left(2\gamma\:\right)\mathrm{sin}\left({\delta\:}_{s}\right)\right)u+\left(\mathrm{cos}\left({\delta\:}_{s}\right)\right)v$$

These Eqs. (9–11) would in principle allow the determination of $\:\gamma\:$ and $\:{\delta\:}_{s}$ at each pixel.

For purely circularly polarised incident light, *I = I*_*p*_, i.e. (1, 0, 0, 1), Eqs. (9–11) simplify to:

12 $$\:{q}^{{\prime\:}}=-\mathrm{sin}\left(2\gamma\:\right)\mathrm{sin}\left({\delta\:}_{s}\right)$$

13 $$\:{u}^{{\prime\:}}=\mathrm{cos}\left(2\gamma\:\right)\mathrm{s}\mathrm{i}\mathrm{n}\left({\delta\:}_{s}\right)$$

14 $$\:{v}^{{\prime\:}}=\mathrm{cos}\left({\delta\:}_{s}\right)$$

From Eq. (14), the value of phase retardation $\:{\delta\:}_{s}$ can be directly determined. This approach provides a relatively simple way for mapping birefringence-induced retardation in a sample. In principle, $\:\gamma\:$ can be determined by the ratio of Eqs. (12) and (13), which gives rise to $\:q{\prime\:}/u{\prime\:}\:=-\mathrm{t}\mathrm{a}\mathrm{n}\left(2\gamma\:\right)$, provided that $\:{\delta\:}_{s}$is not zero. When $\:{\delta\:}_{s}$ is zero, the optical axis orientation $\:\gamma\:$ is no longer defined. For the present study, the focus was exclusively on determining the phase retardation of the samples.

Since the protocol assumes an ideal circularly polarised input, any deviation from perfect circular polarisation introduces an error in the phase retardation measurement. This error is expected to depend on both the polarisation azimuthal angle of the input light and the sample orientation. In our setup, the ellipticity angle was measured at 41°, slightly below the ideal 45° for perfect circular polarisation.

To assess the robustness of the measurement under these conditions, we compared phase retardation images obtained using different angular settings of the circular polariser and varying sample orientations (Figure S4).

To estimate the phase retardation error, a retarder with known retardation was placed over the sample, providing a uniform baseline across the image. From this, the measurement error was estimated to be approximately 1–2%. Details of this validation, along with Figure S5, are provided in Section S.5 of the Supplementary Materials. The figure also demonstrates that phase retardation noise was less than 0.005 radians.

### Image processing

Initial image analysis was performed using Python scripts developed in-house to compute the Stokes parameters, ellipticity angle, polarisation azimuthal angle, and phase retardation. The codes calculated$\:{S}_{DC}$,$\:{S}_{\mathrm{Q}\mathrm{U}1}$, $\:{S}_{\mathrm{Q}\mathrm{U}2}$ and $\:{S}_{\mathrm{v}}$ from images acquired with appropriate intensity modulations which were phase locked to the reference signals. Equation (1) was then applied to obtain the Stokes parameters; Eqs. (3) and (4) were used to derive the ellipticity angle and polarisation azimuthal angle, respectively. Equations (5) and (6) calculated the depolarisation; and Eq. (14) provided the phase retardation. Further image processing was performed using Fiji (ImageJ, USA). Image brightness was adjusted for visualisation and to remove non-specific background, facilitating consistent visual comparison. The background signals have been retained in the Stokes parameter images, as they possess physical significance related to the polarisation states, including the polarisation azimuth angle, ellipticity angle, and degree of depolarisation. In contrast, the non-specific background in the phase retardation images has been removed, and the absolute phase retardation values are presented. Scale bars were added to each panel to indicate spatial dimensions. Intensity images and Q, U, V images were divided by 1,000,000 to avoid long strings of zeros in the colour bar. Figure panels were assembled in Microsoft PowerPoint (Microsoft, USA).

### Statistical analysis

To evaluate the robustness of the quantitative polarimetric measurements, statistical analyses were performed on selected image regions. For each tissue type (placenta, normal skin, and keloid dermis), multiple Regions of Interest (ROIs) of approximately 900 pixels were defined within representative image fields, avoiding artefacts and edge effects. The ROIs (*n* = 10) were square shaped with identical dimensions of 48.6 × 48.6 μm² and were derived from different regions within a single specimen of each tissue type to ensure representative sampling.

From each ROI, values for phase retardation and depolarisation were extracted from the processed polarimetric maps. Shapiro-Wilk tests were performed for evaluating data normality. For each parameter, the mean and standard deviation (SD) were calculated across all ROIs within a given tissue type. Comparisons between groups (e.g., Congo red vs. picrosirius red staining, normal dermis vs. keloid) were performed using two-tailed unpaired t-tests, or Mann-Whitney U where appropriate. All analyses and data visualisation were performed in SPSS (SPSS statistics, IBM, v 29). Results are presented as box plots. *P* values of ≤ 0.05 were considered statistically significant and differences between groups were represented using asterisks or compact letter displays.

## Supplementary Information

Below is the link to the electronic supplementary material.

Supplementary Material 1

## Data Availability

The data generated and/or analysed during the current study are available from the corresponding author on reasonable request.

## Code availability

Custom Python scripts used for image acquisition and analysis, including computation of Stokes parameters, ellipticity, polarisation azimuth, depolarisation, and phase retardation, are available from the corresponding author on reasonable request.
