# Supplementary material for: Polarimetric imaging of collagen in histopathology specimens: an investigation of congo red and picrosirius red-stained placenta and skin
Source: Sci Rep. 2026 Mar 6;16:12441. doi: 10.1038/s41598-026-37711-8 (PMC13083921; doi:10.1038/s41598-026-37711-8)
Supplement: Supplementary file 1 — Supplementary Material 1 [file 41598_2026_37711_MOESM1_ESM.docx]

# Polarimetric imaging of collagen in histopathology specimens - an investigation of Congo red and picrosirius red-stained placenta and skin

# Georgia Mappa^1^, Pika Miklavc^2^, Michele Cummings^1^, Richard Oliver^3^, Harriet Pyrah^1^, Clare Freer^1^, Huda Alzahrani^4^, Tiehan H Shen^2^* and Nicolas Orsi^1^*.

# Supplementary Materials

## S.1 Linearity of the imaging system

The methodology of the Stokes parameter determination is based on the basic assumption that the detection system has a linear response to the input intensity. To verify it for the camera system, the intensity detected by the camera was plotted against that detected by the Si photo diode known to have a linear behaviour. The linear relationship between the two set of signals in Figure S1 illustrated that the camera, as required, had a linear response in the experimental setting.


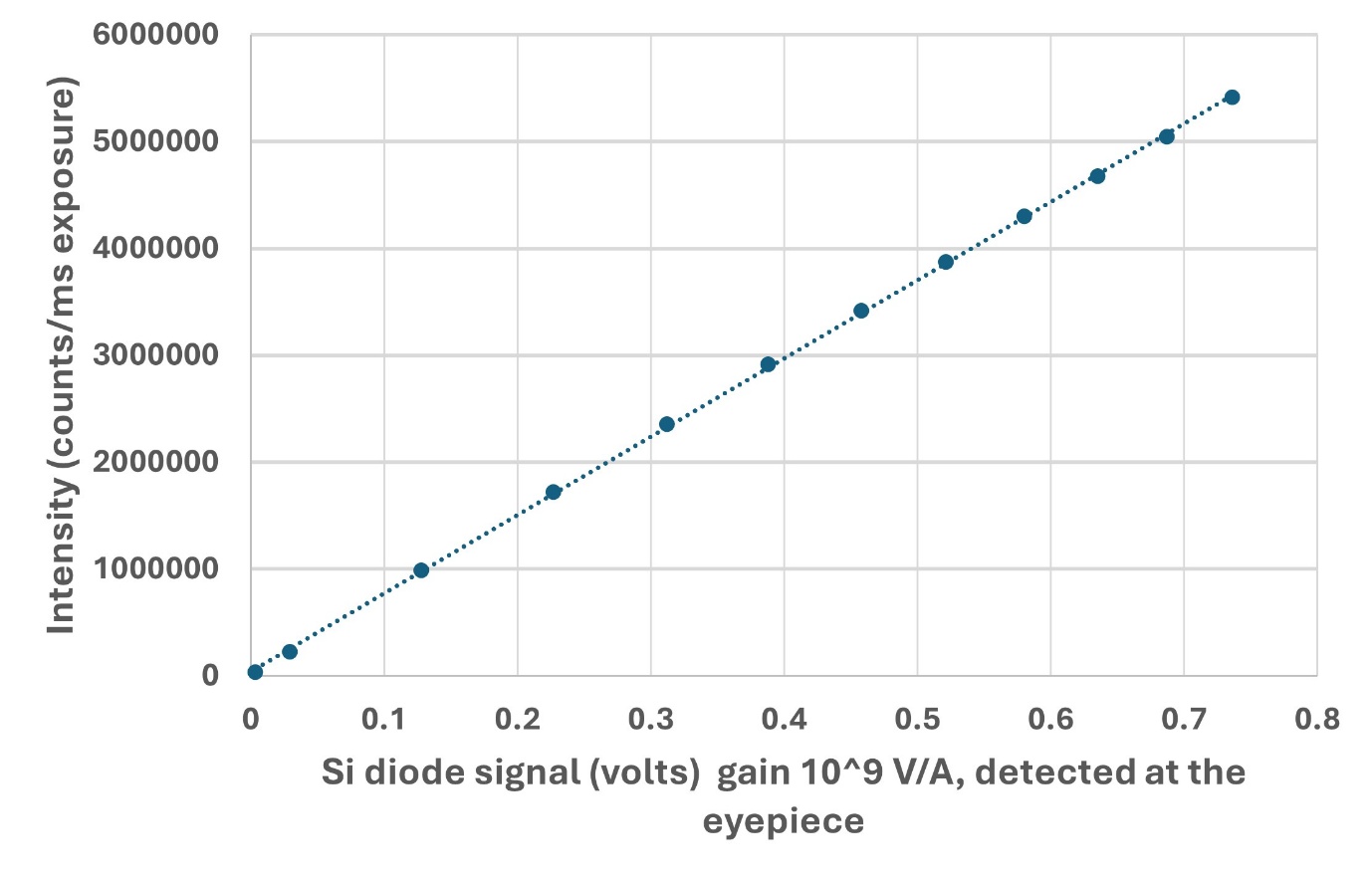


**Figure S1:** The intensity detected by the camera versus that of the Si photo diode showing a linear relationship. The gain of the Si photo diode is 10^9^ V/A.

## S.2 Polarimeter calibration

As an example, Figure S2 shows typical calibration results obtained using both elliptical and linear polarisation states for the 530 nm LED source. Figure S2 (a) shows the data and the theoretical values for polarisation states generated by rotating a phase retarder which is a quarter-wave plate for the wavelength of 670nm (retardance of 167.5nm). Since the retarder is not a quarter-wave plate for the LED source, it generated elliptical polarised light with a maximum ellipticity angle of about 33°, hence the maximum *v* value is only about 0.915. Figure S2 (b) shows the data and the theoretical values for polarisation states generated by rotating a linear polariser. The residual in azimuthal angle of linearly polarised light was less than 0.003 radian and that of the ellipticity angle for elliptically polarised light was also of similar size. This was comparable to the precision for manually setting the angles in the calibration process for the present experimental setup.


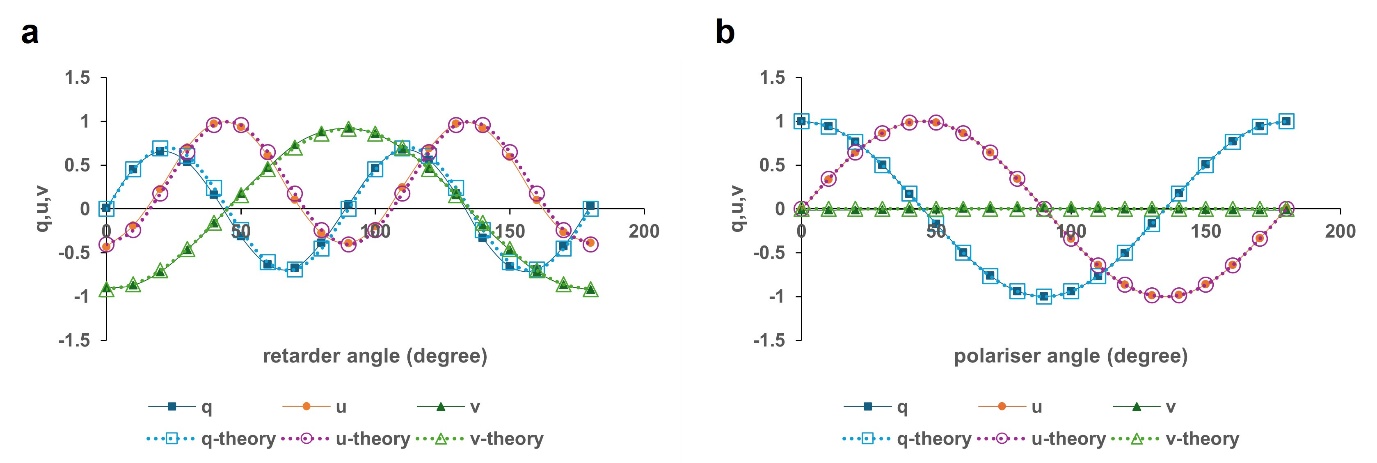


**Figure S2:** Calibration data for 530nm LED source. The calibration algorithm determines the non-zero elements in the matrix *K* using nonlinear optimisation. The results are shown in the normalised Stokes parameters where *q* = *Q*/*I*, *u* = *U*/*I* and *v* = *V*/*I*. (a) theoretical and experimental curves for polarisation states generated by rotating a phase retarder. (b) theoretical and experimental curves for polarisation states generated by rotating a linear polariser. Note that for different linear polarisation states, the circular polarisation component is expected to be zero, as confirmed by the zero *v* values in (b).

## S.3 Polarimetric Placental Tissue Profiling

Polarimetric imaging provided quantitative insights into the structural organisation of collagenous components within placental tissue. Figure S3 (a)-(c) presents the spatial distribution of the Stokes parameters *Q*, *U* and *V*, respectively, for a Congo red-stained placental section under nearly circularly polarised illumination, collectively (with *I*) describe the full polarisation state at each pixel. For comparison, images from a sequential placental section stained with picrosirius red are shown in Figure S3 (d)-(f), showing the spatial distribution of the Stokes parameters *I*, *Q*, *U* and *V*, respectively.


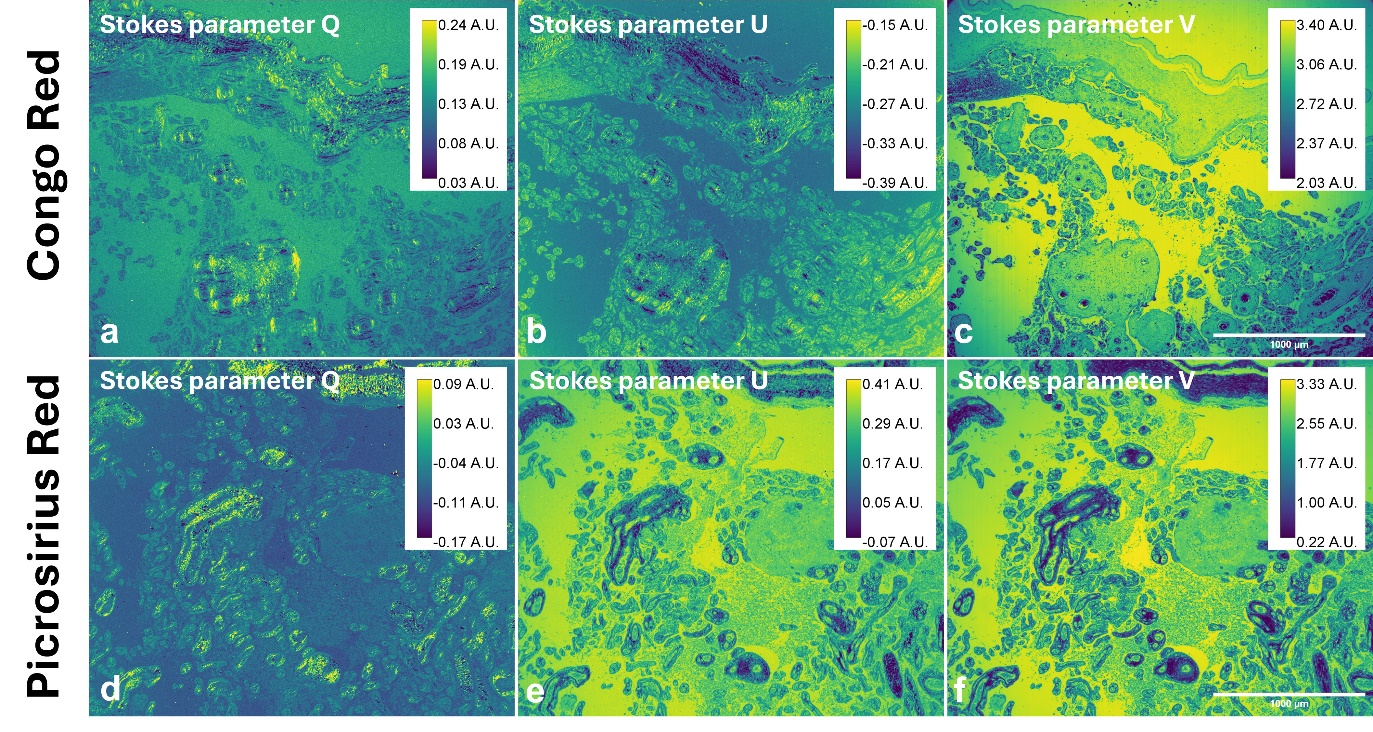


**Figure S3:** Representative polarimetric imaging of a Congo red-stained placenta section under nearly circularly polarised light illumination using a 4x objective lens. (a) Stokes parameter *Q*: intensity difference of linear polarisation along two orthogonal axes), (c) *U*: intensity difference of linear polarisation along two orthogonal axes rotated 45º with respect to that of *Q*, and (d) *V*: intensity difference between the left and right hand circular polarisation, with *I* collectively pertaining the full polarisation state of light at the sample. For comparison, corresponding polarimetric images of a sequential placental section stained with picrosirius red and imaged under identical conditions are shown in (d)-(f): (d) Stokes parameter *Q*, (e) *U* and (f) *V*. Scale bars: 1000 µm. A.U.: Arbitrary Units

## S.4 The robustness in determining the phase retardance

The protocol for the determination of the phase retardance, $\delta_{s}$, assumes that the polarisation state of the incoming light is perfectly circularly polarised with the ellipticity angle being precisely 45°. In the present study a nearly circular polarised light source was used, with an ellipticity angle of about 41°. The non-ideal polarisation state would be expected to introduce an angular variation with sample or circular polariser rotation. Imperfect optical alignment of the microscope optics would also introduce similar variation. To experimentally verify the robustness of $\delta_{s}$, images were taken with either the sample being rotated (about 30°) or the circular polariser being rotated (also about 30°). Figure S4 compares the $\delta_{s}$ images under those rotations. For the Congo red stained placenta sample: Figure S4(a) shows the sample at its normal orientation and the circular polariser at its normal orientation; Figure S4(b), the sample at its normal orientation and the circular polariser rotated; Figure S4(c), the sample rotated and the circular polariser at its normal orientation, where the image is re-orientated for easier comparison. Similarly, for the picrosirius red stained placenta sequential section: Figure S4(d) shows the image of the sample without either sample or circular polariser rotation; Figure S4(e) with circular polariser rotation and Figure S4(f) with sample rotation only, where the image is also re-orientated for easier comparison. The images show broadly their similarity to one another, indicating that the methodology was sufficiently robust even with a nearly circular polarised light of an ellipticity angle of about 41°.

The brightest regions have a typical retardance value of about 0.03 radians for the Congo red stained sample and 0.04 radians for the Picrosirius red stained sample.


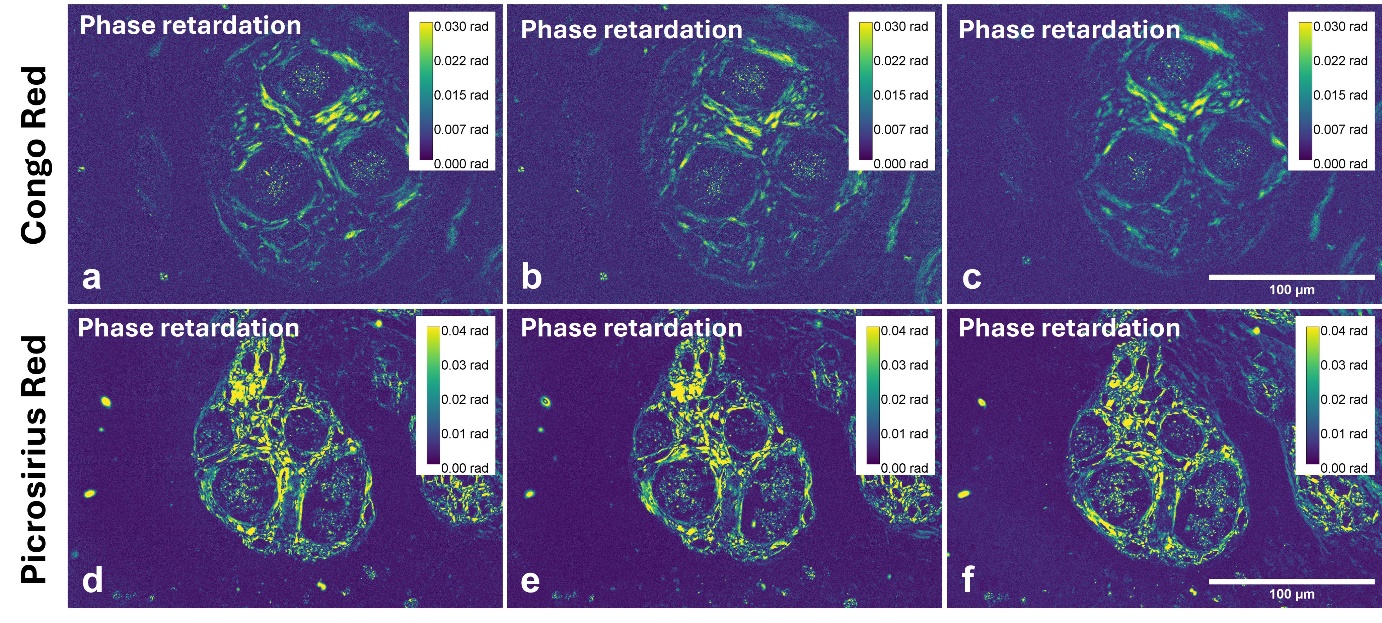


**Figure S4:** Effect of rotations of circular polariser (CP) or sample (S), where (0) denotes no rotation and (R) denotes rotation: (a) Congo red, CP(0), S(0), (b) Congo red, CP (R), S (0), (c) Congo red, CP (0), S (R), (d) Picrosirius red, CP (0), S (0), (e) Picrosirius red, CP (R), S (0), (f) Picrosirius red, CP (0), S (R). Scale bars: 100 µm.

## S.5 The baseline in the phase retardance image

To illustrate the accuracy of the phase retardance values, a retarder with a known phase retardance of 1.986 radians was inserted between the sample and the condenser. This would add a baseline in the phase retardance image. Figure S5 shows typical results with the retarder inserted (Figure S5(a) and (b)), with the retarder rotated by about 20° (Figure S5(c) and (d)) and without the retarder (Figure S5(e) and (f)).

Figure S5(b) shows an average baseline value of 2.114 radians, whilst Figure S5(d) 2.015 radians. The baseline value without the retarder (Figure S5(f)) is 0.106 radians. Subtracting the latter value, the experimentally determined phase retardance for the retarder was either 2.008 radians (Figure S5(b)) or 1.909 radians (Figure S5(d)). The variation may be attributed to the non-ideal circular polarisation illumination as discussed previously in section S.4. Compared with the known value of 1.986 radians, the error in the experimental value is estimated to be about 1 to 2 percent. From the line profiles, the noise in the phase retardance is less than 0.005 radians, limiting the sensitivity of phase retardance to that level. However, noise may be reduced further if a longer imaging acquisition time is used.


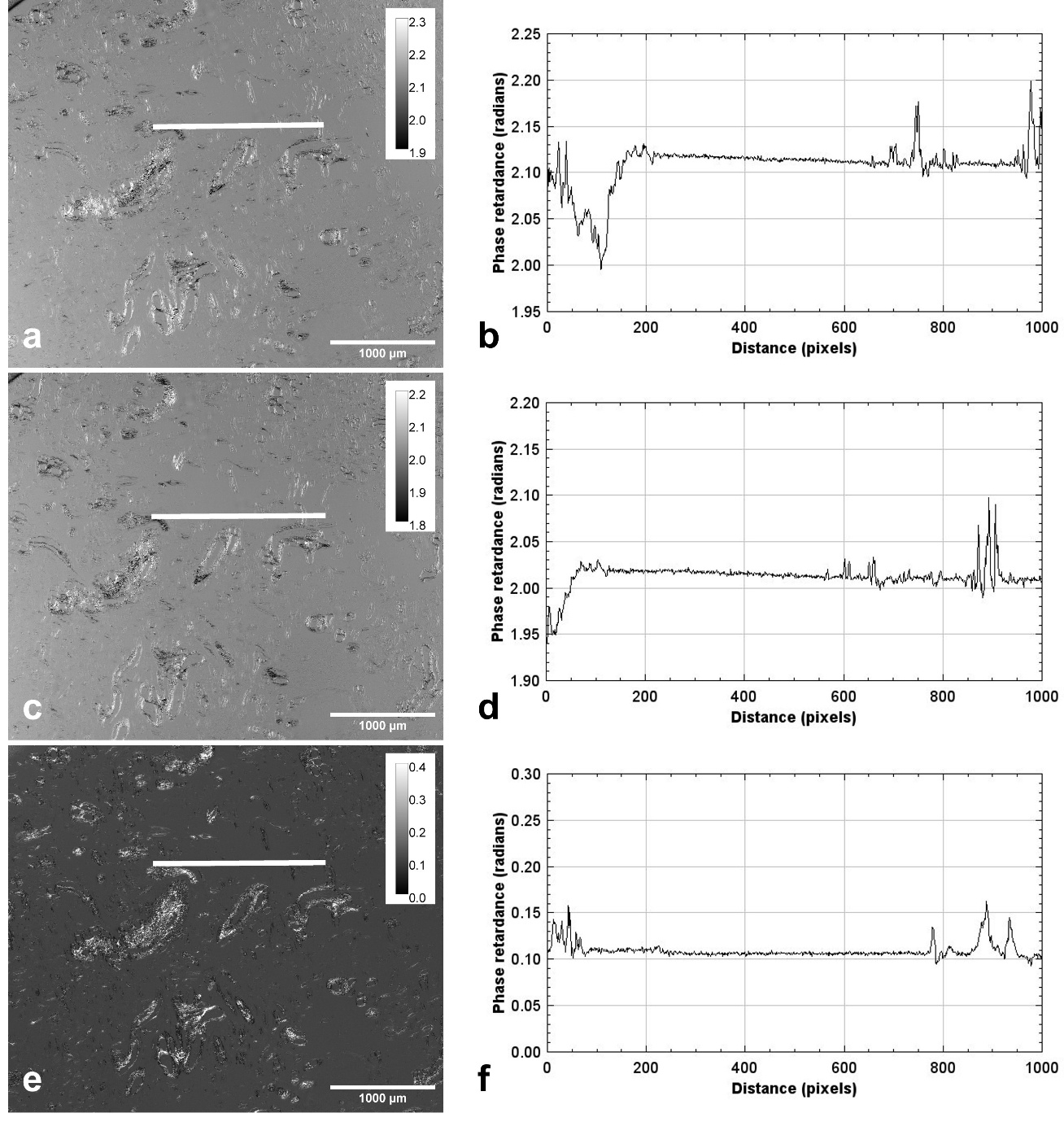


**Figure S5:** Picrosirius red stained placenta sample: (a) the image with a phase retarder inserted over the sample. The long white line indicates the location where the line profile was taken; (b) the phase retardance profile along the line in (a); (c) the image with the phase retarder rotated by about 20° with the line indicates the location of the line profile; (d) the phase retardance profile along the line in (c); (e) the image with the phase retarder removed with the line marking the location of the line profile; (f) the phase retardance profile along the line indicated in (e). Scale bars: 1000 µm.

## S.6 Immunohistochemistry

To confirm that the distribution of collagen matched the birefringence signal acquired with QPM, immunohistochemistry was carried out. Antigen retrieval was performed on tissue sections with Trilogy (Sigma-Aldrich, UK) according to the manufacturer’s instructions followed by quenching endogenous enzyme activity in Bloxall (Vector Laboratories, UK) for 15 min. Sections were then incubated for 20 minutes with 2.5% Normal Horse Serum at room temperature and then overnight at 4 °C with anti-collagen (1:200; Thermofisher). Following washing, slides were incubated for 30 min in either horse anti-mouse or horse anti-rabbit IgG polymer reagent (Vector). Antibodies were visualised using diaminobenzidine (DAB, Vector), or Red Substrate (Vector). Slides were counterstained with haematoxylin (Sigma-Aldrich), cleared and mounted (Figure S6).

**
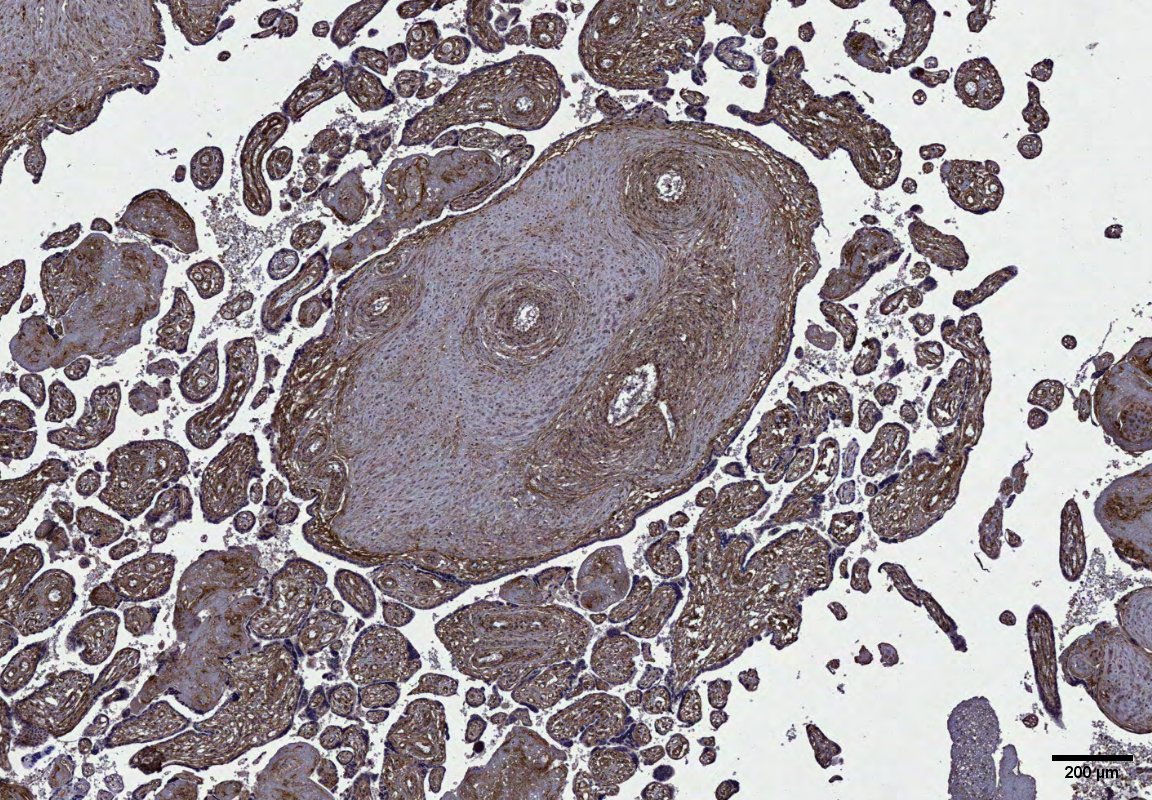
**

**Figure S6:** Representative image of placenta stained with pan-collagen antibody. Note the concentric distribution of collagen fibres surrounding villous vessels as identified with QPM. Scale bar: 200 μm.

## S.7 Statistical analysis – Graphical representation

To evaluate the robustness of our findings, multiple Regions of Interest (ROIs) across each tissue type (e.g., keloid, normal dermis) were defined. The ROIs were used to calculate the mean and standard deviation for phase retardation and depolarisation. Statistical analysis was performed to assess the significance of observed differences. Graphical representations of all outputs are provided in Figure S7.1 – 7.3.


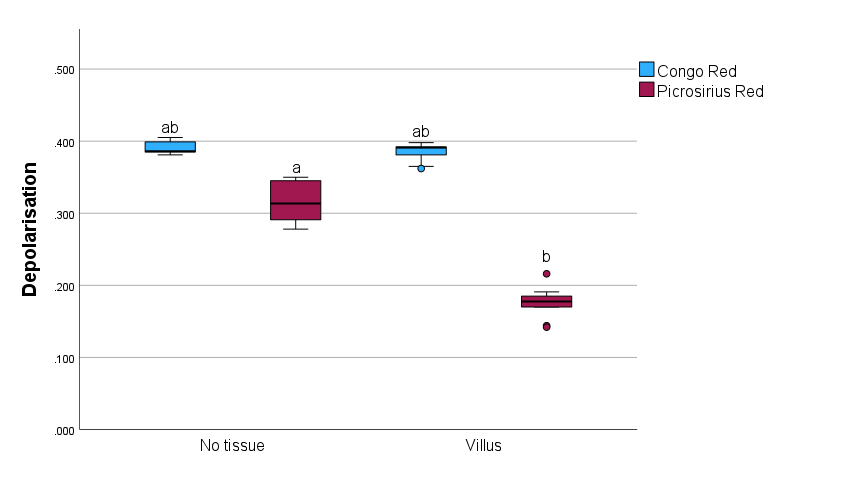

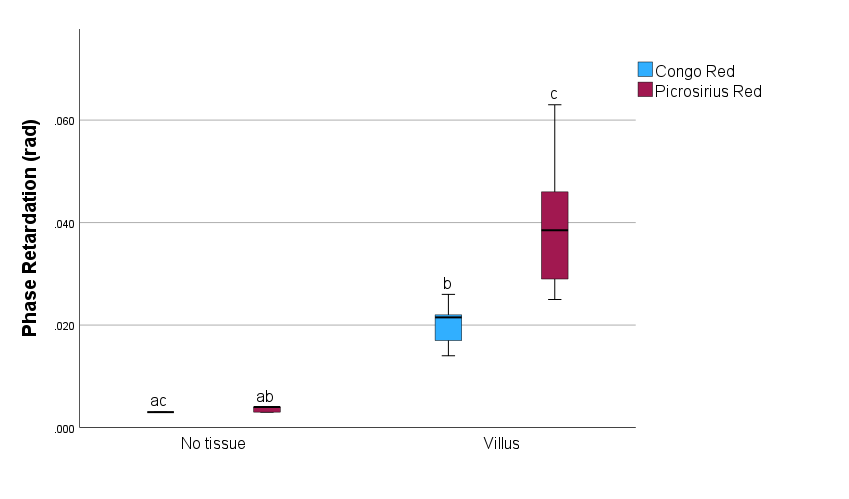


**A**

**B**

**Figure S7.1:** Placental (A) depolarisation and (B) phase retardation. Measurements were extrapolated from representative ROIs for all groups. Data are presented as boxplots and non-shared letters denote statistically significant differences between groups (*P*<0.05).


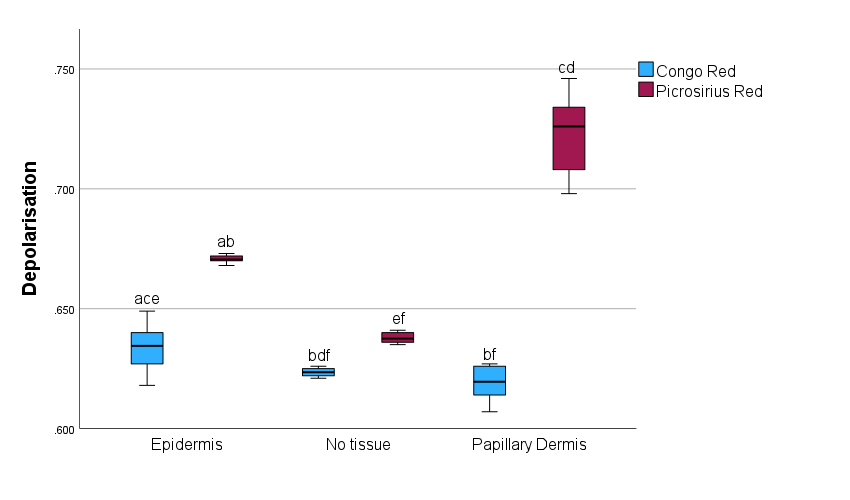

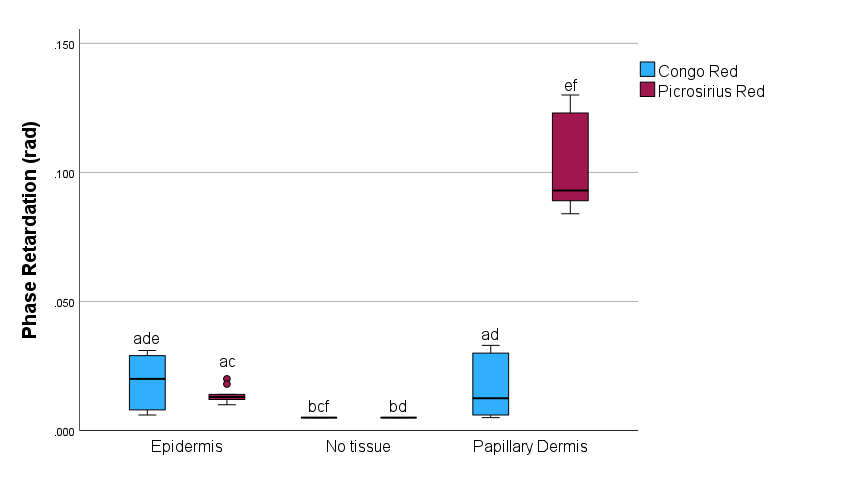


**A**

**B**

**Figure S7.2:** Skin (A) depolarisation and (B) phase retardation. Data were obtained from defined ROIs across different areas and are presented as boxplots. Non-shared letters denote statistically significant differences between groups (*P*<0.05).


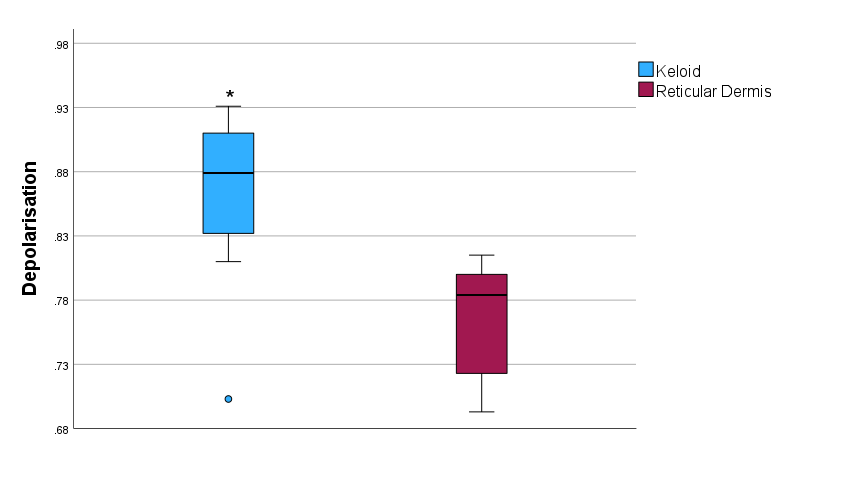

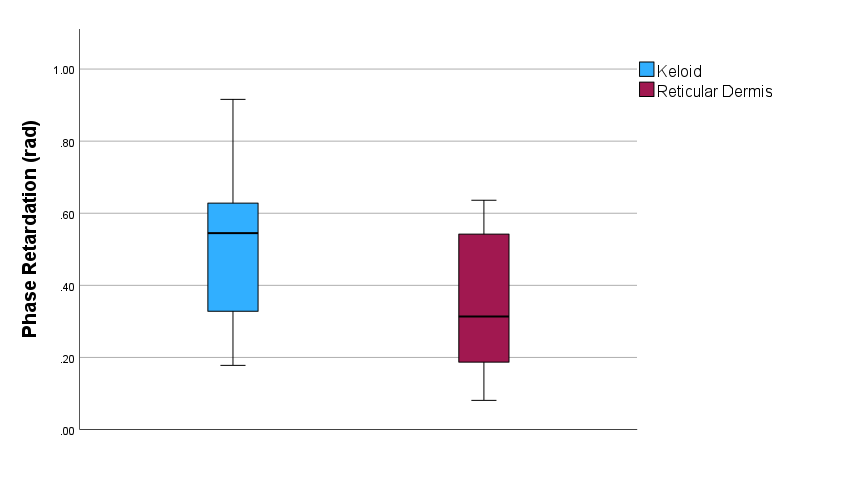


**A**

**B**

**Figure S7.3:** Reticular dermis and keloid (A) depolarisation and (B) phase retardation. Measurements were extrapolated from representative ROIs for all groups. Data are presented as boxplots and asterisks denote statistically significant differences between groups (*P*<0.05).
